# Short-term effects of announcing revised lower risk national drinking guidelines on related awareness and knowledge: a trend analysis of monthly survey data in England

**DOI:** 10.1136/bmjopen-2016-013804

**Published:** 2016-12-01

**Authors:** John Holmes, Jamie Brown, Petra Meier, Emma Beard, Susan Michie, Penny Buykx

**Affiliations:** 1 Sheffield Alcohol Research Group, School of Health and Related Research (ScHARR), University of Sheffield, Sheffield, UK; 2 UK Centre for Tobacco and Alcohol Studies (UKCTAS), Nottingham, UK; 3 Department of Clinical, Educational and Health Psychology, University College London, London, UK; 4 Cancer Research UK Health Behaviour Research Centre, University College London, London, UK

**Keywords:** Alcohol, Drinking guidelines, Health promotion, Evaluation, Trend analysis

## Abstract

**Objectives:**

To evaluate short-term effects of publishing revised lower risk national drinking guidelines on related awareness and knowledge. To examine where drinkers heard about guidelines over the same period.

**Design:**

Trend analysis of the Alcohol Toolkit Study, a monthly repeat cross-sectional national survey.

**Setting:**

England, November 2015 to May 2016.

**Participants:**

A total of 11 845 adults (18+) living in private households in England.

**Intervention:**

Publication of revised national drinking guidelines in January 2016 which reduced the male guideline by approximately one-third to 14 units per week.

**Measurements:**

Whether drinkers (1) had heard of drinking guidelines (awareness), (2) stated the guideline was above, exactly or below 14 units (knowledge) and (3) reported seeing the stated guideline number of units in the last month in each of 11 locations (exposure). Sociodemographics: sex, age (18–34, 35–64, 65+), social grade (AB, C1C2, DE). Alcohol consumption derived from graduated frequency questions: low risk (<14 units/week), increasing/high risk (14+ units/week).

**Results:**

Following publication of the guidelines, the proportion of drinkers aware of guidelines did not increase from its baseline level of 85.1% (CI 82.7% to 87.1%). However, the proportion of male drinkers saying the guideline was 14 units or less increased from 22.6% (CI 18.9% to 26.7%) in December to 43.3% (CI 38.9% to 47.8%) in January and was at 35.6% (CI 31.6% to 39.9%) in May. Last month exposure to the guidelines was below 25% in all locations except television/radio where exposure increased from 33% (CI 28.8% to 36.2%) in December to 65% (CI 61.2% to 68.3%) in January. Awareness and knowledge of guidelines was lowest in social grade DE and this gap remained after publication.

**Conclusions:**

Publication of new or revised lower risk drinking guidelines can improve drinkers’ knowledge of these guidelines within all sociodemographic groups; however, in the absence of sustained promotional activity, positive effects may not be maintained and social inequalities in awareness and knowledge of guidelines are likely to persist.

**Strengths and limitations of this study**

- Low-risk drinking guidelines are published by governments or health authorities in most high-income countries but they are rarely evaluated and little robust evidence is available evaluating their effects on outcomes of interest.
- To the authors’ knowledge, this study is the first internationally to use prospective, high-frequency survey data to examine the short-term effects of publishing new or revised drinking guidelines.
- Monthly data allowed for examination of how short-term effects emerge and decay after a major component of public health information is announced and from what sources the public heard about this information at different time points.

## Introduction

In January 2016, the UK's Chief Medical Officers published proposed revisions to the country's lower risk drinking guidelines.^1^ The previous guidelines were published in 1995 and recommended not regularly consuming more than 3–4 units of alcohol a day for men and 2–3 units a day for women (1 UK unit=10 mL/7.9 g ethanol). Regularly was defined as not drinking that amount every day or nearly every day. A review of these guidelines was announced in 2012 as a major component of the UK Government's Alcohol Strategy.^2^ The review was particularly informed by a UK parliamentary report which noted increased evidence of a causal relationship between alcohol consumption and cancer and increasing scepticism regarding purported benefits of moderate drinking for cardiovascular health.^3^

The new guidelines were developed between 2013 and 2016 by an expert committee who reviewed existing evidence and commissioned new epidemiological modelling and studies of public attitudes.^1^ The epidemiological modelling played an important role in the committee's decision-making as it estimated the levels and patterns of alcohol consumption which, if adopted by the entire UK population, would correspond to each of two definitions of low-risk drinking: having a risk equivalent to that of current UK abstainers and having a 1% lifetime risk of dying due to alcohol.^4^ These definitions were previously used in guideline review processes in Canada and Australia and the latter definition has also been used in a recent analysis of alcohol-related risks in the European Union.^5–7^ Thus, the new guidelines contain three major changes: (1) from a daily to weekly guideline; (2) equalising the guidance for men and women; (3) a reduction in the guideline for men which was often interpreted as 21 units per week—a legacy of the pre-1995 guidance. In announcing the new guidelines, the Chief Medical Officers and the UK Government also placed significant emphasis on there being ‘no safe level’ of alcohol consumption with regard to cancer risks and downplayed benefits for cardiovascular health.^8^

Although drinking guidelines are published in at least 37 countries,^9^ there is little evidence documenting their effects or how those effects emerge and decay following promotional activity.^10–12^ The few published studies suggest that promotional activity may improve awareness and knowledge of the guidelines without reducing consumption. However, such claims are typically based on studies with limited potential for causal inference; for example, studies using cross-sectional surveys repeated at 1-year intervals.^13–20^

To date, there has been no official large-scale promotional campaign for the new UK guidelines. However, the announcement was a lead story for national news outlets and attracted substantial commentary in subsequent weeks, some of which was highly critical. Many health websites and other promotional materials which mention the guidelines have been updated but alcoholic drink labels remain unchanged, even though ∼80% of alcoholic product labels in the UK include the drinking guidelines.^21^

This study uses monthly cross-sectional survey data to assess the size and duration of short-term effects of announcing new UK lower risk drinking guidelines on drinkers' guideline-related awareness and knowledge. In addition, it investigates trends in drinkers' sources of information about the guidelines and variation in changes in awareness and knowledge by age, sex, socioeconomic status and alcohol consumption level.

## Methods

### Data

Data come from the Alcohol Toolkit Study (ATS), a monthly repeat cross-sectional survey which began in March 2014 and collects data from nationally representative samples of ∼1600 adults each month living in private households in England. Since November 2015, the ATS has included questions relating to the drinking guidelines, and this analysis uses data from the 7 months between November 2015 and May 2016 (the most recently available month). January data were collected in the week after publication of the revised guidelines.

The full ATS methods are described elsewhere.^22^ Briefly, monthly samples are collected as part of a wider omnibus survey by the research agency Ipsos Mori using in-home computer-assisted interviewing. The survey uses a hybrid between random location sampling and quota sampling whereby England is split into 171 356 areas containing ∼300 households. Areas are then allocated to interviewers based on stratified random sampling with strata being area-level geographic and socioeconomic profiles. Interviews are conducted within the randomly selected areas until quotas based on factors influencing the probability of being at home are filled (eg, employment status, age, gender). Prevalence data are weighted using an iterative sequence of weighting adjustments whereby separate nationally representative target profiles are set for gender, working status, children in the household, age, social grade and region. This process is then repeated until all variables match the specified targets.

Analyses here focused on drinkers who were identified via the AUDIT questionnaire, a widely used screening instrument for problem drinking which has good validity, high internal consistency and good test–retest reliability across gender, age and cultures.^22^ ^23^ Those who responded that they never drink were classed as non-drinkers.

### Measures

#### Awareness

Awareness of guidelines among drinkers was measured using the question: ‘Before this interview, have you ever heard of there being a recommended maximum number of alcohol units people should drink in a day or a week? This is sometimes known as a “drinking guideline”’. Responses were dichotomised as yes or no. The concept of units was explained during the AUDIT questionnaire which was administered earlier in the survey.

#### Knowledge

Knowledge of the guideline among those who had heard of the concept was measured using the question: ‘How many units per day or per week is the drinking guideline for males/females?’. Participants were asked about their own sex only and allowed to respond in units per week or per day. For this analysis, responses were either trichotomised as more than, exactly or below 14 units per week or 2 units a day or dichotomised as above versus exactly or below 14 units per week or 2 units per day (hereafter 14 units per week or 14 units for brevity). These classifications were used to test whether announcing the new guidelines increased the proportion of people saying the guideline was 14 units per week.

#### Exposure

To assess where people see or hear about the guideline figure they gave, those who gave a figure were asked which of a list of places they had seen, read or heard about it in the last month (table 1). Responses were dichotomised as yes or no and there was no limit on the number of places participants could say ‘yes’ to.

**Table 1:** Trends in main outcome measures by survey month*

	November 2015	December 2015	January 2016	February 2016	March 2016	April 2016	May 2016
Drinker status: base—all respondents (N)	1689	1660	1712	1674	1679	1711	1720
Drinkers	71.2	65.9	66.1	65.2	67.7	66.6	68.0
	(67.8 to 72.5)	(63.4 to 68-2)	(63.7 to 68.5)	(62.6 to 67.7)	(65.3 to 70.1)	(64.2 to 68.9)	(65.7 to 70.2)
Awareness: base—all drinkers (N)	1102	1035	1103	1037	1109	1098	1141
Heard of guidelines	85.1	87.1	88.6	88.6	86.5	85.9	88.4
	(82.7 to 87.1)	(84.8 to 89.0)	(86.5 to 90.4)	(86.4 to 90.5)	(84.2 to 88.5)	(83.6 to 87.9)	(86.4 to 90.2)
Knowledge of new guideline: base—all drinkers (N)	1104	1035	1111	1040	1119	1100	1143
Below 14 units per week	18.3	17.9	**23.4**	19.8	19.3	19.9	19.9
	(15.8 to 21.1)	(15.5 to 20.6)	**(20.6** to **26.1)**	(17.2 to 22.6)	(16.8 to 21.8)	(17.5 to 22.6)	(17.5 to 22.5)
14 units per week	19.4	20.6	**29.0**	**27.1**	24.4	24.7	**26.3**
	(16.8 to 22.2)	(18.1 to 23.5)	**(26.0** to **31.8)**	**(24.1** to **30.2)**	(21.7 to 27.0)	(22.1 to 17.6)	**(23.7** to **29.1)**
Above 14 units per week	32.0	33.2	**22.1**	29.7	27.7	**27.1**	**25.5**
	(29.0 to 35.1)	(30.2 to 36.4)	**(19.4** to **24.7)**	(26.6 to 32.8)	(24.7 to 30.4)	**(24.5** to **30.0)**	**(23.0** to **28.3)**
Not aware of drinking guidelines	14.9	13.0	11.4	11.4	13.5	14.1	11.6
	(12.8 to 17.3)	(11.0 to 15.2)	(9.5 to 13.4)	(9.5 to 13.4)	(11.4 to 15.7)	(12.1 to 16.3)	(9.8 to 13.6)
Aware of but doesn't know guideline	15.4	15.3	14.2	12.1	15.1	14.1	16.7
	(13.1 to 18.1)	(13.1 to 17.8)	(12.7 to 17.0)	(10.3 to 14.7)	(13.6 to 18.1)	(12.1 to 16.5)	(14.6 to 19.2)
Exposure in last month: base—drinkers who gave a figure for the guideline (N) *Multiple responses permitted*	771	742	822	786	804	804	856
Product labels	20.8	19.4	17.8	14.3	21.0	18.7	21.8
	(17.8 to 24.2)	(16.5 to 22.7)	(15.1 to 20.9)	(11.8 to 17.2)	(18.1 to 24.2)	(16.0 to 21.8)	(19.0 to 24.9)
TV/radio	35.8	32.5	**64.8**	**54.0**	**50.9**	**44.2**	**47.2**
	(32.1 to 39.7)	(28.9 to 36.2)	**(61.2** to **68.3)**	**(50.1** to **57.9)**	**(47.2** to **54.6)**	**(40.6** to **47.9)**	**(43.7** to **50.8)**
Newspapers/magazines	16.5	15.0	**24.3**	**22.9**	20.5	17.7	19.8
	(13.8 to 19.7)	(12.4 to 18.0)	**(21.2** to **27.6)**	**(20.0** to **26.3)**	(17.7 to 23.6)	(15.2 to 20.6)	(17.2 to 22.7)
Websites/social media	5.7	6.3	7.5	5.3	8.8	6.4	8.4
	(4.2 to 7.8)	(4.6 to 8.7)	(5.6 to 9.8)	(3.9 to 7.2)	(6.8 to 11.4)	(4.8 to 8.5)	(6.6 to 10.6)
Shops/supermarkets	8.6	8.0	6.7	7.7	7.9	5.2	8.2
	(6.6 to 11.0)	(6.2 to 10.4)	(5.2 to 8.8)	(6.0 to 10.0)	(6.2 to 10.1)	(3.8 to 7.0)	(6.4 to 10.3)
Pubs/bars/restaurants	13.2	12.8	11.3	10.1	11.2	8.6	10.8
	(10.8 to 16.1)	(10.4 to 15.6)	(9.2 to 13.9)	(8.0 to 12.6)	(9.0 to 13.8)	(6.7 to 10.9)	(8.1 to 12.5)
At place of work/study	7.1	6.0	7.2	8.0	8.1	6.6	8.6
	(5.1 to 9.6)	(4.4 to 8.1)	(5.4 to 9.5)	(6.1 to 10.5)	(6.3 to 10.4)	(4.9 to 8.7)	(6.8 to 10.8)
Talking to health professionals	10.8	6.9	6.9	5.3	8.1	7.0	6.7
	(8.7 to 13.5)	(5.3 to 9.1)	(5.3 to 9.1)	(3.9 to 7.4)	(6.3 to 10.3)	(5.4 to 9.1)	(5.2 to 8.6)
Posters/booklets at health service	11.5	10.6	9.6	9.0	12.0	8.3	10.5
	(9.2 to 14.2)	(8.5 to 13.2)	(7.6 to 12.1)	(7.1 to 11.4)	(9.8 to 14.6)	(6.5 to 10.6)	(8.6 to 12.9)
Talking to friends/family/colleagues	7.6	5.1	**9.2**	8.3	**8.9**	6.6	**9.6**
	(5.8 to 9.9)	(3.7 to 7.1)	**(7.3** to **11.5)**	(6.5 to 10.7)	**(7.1** to **11.1)**	(5.0 to 8.6)	**(7.7** to **11.9)**
Other	1.5	1.2	0.3	0.6	0.4	0.5	0.3
	(0.8 to 2.8)	(0.6 to 2.4)	(0.1 to 1.2)	(0.3 to 1.5)	(0.1 to 1.1)	(0.1 to 1.5)	(0.1 to 1.0)
None of the above	26.4	32.3	**7.4**	**13.3**	**15.5**	**23.3**	**15.0**
	(23.1 to 30.0)	(28.7 to 36.0)	**(5.7** to **9.7)**	**(10.9** to **16.2)**	**(13.0** to **18.4)**	**(20.3** to **26.7)**	**(12.7** to **17.7)**

*All figures are percentages with 95% CIs in parentheses unless otherwise stated. Bold text indicates significant differences compared with December 2015 based on 95% CIs.

#### Sociodemographic and drinking

The following characteristics were assessed: sex, age (18–34, 35–64, 65+) and social grade which is an occupation-based measure of socioeconomic status, trichotomised here as AB (higher and intermediate managerial, administrative or professional occupations), C1C2 (supervisory, clerical, junior managerial, administrative and professional occupations or skilled manual occupations) and DE (semiskilled or unskilled occupations and unemployed).

Alcohol consumption was measured via a graduated frequency approach.^24^ ^25^ Drinkers were asked the maximum amount of alcohol they consumed on a single day in the past 4 weeks and how many units this was. They were then asked on how many days they consumed this amount and on how many days they consumed progressively decreasing numbers of units below this maximum (eg, 31–40, 21–30, 16–20, 11–15, 8–10, 5–7, 3–4, 1–2). The number of days consuming each quantity was multiplied by that quantity (with midpoints used for ranges) and the sum of these multiples was divided by four to give a measure of average weekly consumption. This measure was dichotomised as low risk (<14 units per week) versus increasing/high risk (14+ units per week).

### Analysis

Descriptive analyses are used to examine change in outcome measures compared with December 2015, the last month before new guidelines were announced. Variation between subgroups in exposure to guidelines for the whole time period is also examined descriptively. All analyses are based on weighted survey data and cases are not excluded if they have missing data on some variables. Further analyses presented in the online appendix test for subgroup differences in time trends for the outcome measures using unweighted binary and multinomial regression models with interaction effects between survey month and subgroup characteristics. These analyses lead to identical conclusions and the simpler descriptive analyses are preferred here for the benefit of the reader. All analyses were conducted in Stata SE V.12.1.

10.1136/bmjopen-2016-013804.supp1 Supplementary appendix

### Ethics

Informed consent is given verbally by ATS participants after interviewers explain the study and give assurance that it is being conducted in line with the Market Research Society Code of Conduct.

## Results

In December, 87% of drinkers said they had heard of drinking guidelines (table 1). Despite substantial news coverage around the announcement, this figure did not increase significantly in January and 11% of drinkers said they were unaware of drinking guidelines in that month.

In contrast, there was a change in knowledge of the guideline following the announcement. In December, 33% of drinkers thought the guideline was above 14 units per week and this fell significantly to 22% in January. Conversely, the proportion of drinkers saying the guideline was exactly 14 units increased significantly from 21% to 29%. In the absence of sustained promotional activity, these effects on drinkers' knowledge were not sustained and the proportion of drinkers stating the guideline was 14 units per week fell to 27% in February and 24% in March. There was some evidence of a secondary increase in knowledge emerging gradually from March onwards, but further data points are required to confirm this.

Among drinkers who gave a figure for the guidelines, 32% reported no exposure to this figure in December but this dropped to 7% in January and remained low at 15% in May (table 1). TV and radio were the most common contexts to hear about the guidelines and the proportion who had done so in the last month increased significantly from 33% in December to 65% in January. Exposure to guidelines in newspapers and magazines also increased significantly between December and January, from 15% to 24%. In both media, exposure declined in subsequent months. Exposure may also have increased after December through talking to friends, family and colleagues, but this increase is small and it is unclear whether it is a real change or a result of comparing against an unusual low in December. In all other contexts, recent exposure to drinking guidelines was low and did not increase significantly in January.

When comparing awareness across sociodemographic groups, a majority of drinkers in all groups were aware of guidelines at all time points; however, there were significant differences in awareness by social grade with only 70% of those in social grade DE aware of guidelines in December compared with 89% in grade C1C2 and 93% in grade AB (table 2). This significant difference remained after the announcement in January and in subsequent months.

**Table 2:** Trends in main outcome measures within sociodemographic groups by survey month*

	November 2015	December 2015	January 2016	February 2016	March 2016	April 2016	May 2016
Unweighted number of cases (N)							
Female	805	820	848	817	823	862	871
Male	884	840	871	860	866	850	850
16–34	546	522	505	518	502	521	522
35–64	759	738	759	725	784	706	796
65+	384	400	455	434	403	485	403
Social grade AB	332	376	416	366	371	420	420
Social grade C1C2	893	832	876	856	845	816	896
Social grade DE	464	452	427	455	473	476	406
Non-drinker	585	625	608	637	570	612	578
Low risk (<14 units per week)	677	644	744	648	675	675	702
Increasing/high risk (14+units per week)	178	189	189	195	200	232	200
Per cent of sample who are drinkers							
Female	66.9	61.9	62.3	62.7	63.2	60.9	64.8
	(63.3 to 70.2)	(58.4 to 65.3)	(58.6 to 65.5)	(58.6 to 65.8)	(59.2 to 66.1)	(57.5 to 64.3)	(61.5 to 68.0)
Male	73.8	69.9	70.4	68.2	73.0	72.5	71.3
	(70.5 to 76.8)	(66.4 to 73.2)	(66.9 to 73.6)	(64.6 to 71.6)	(69.7 to 76.1)	(69.2 to 75.6)	(68.1 to 74.4)
16–34	60.1	57.9	60.8	56.2	64.5	60.9	61.2
	(55.6 to 64.6)	(53.3 to 62.4)	(56.1 to 65.3)	(51.4 to 60.8)	(60.0 to 68.8)	(56.5 to 65.1)	(56.8 to 65.5)
35–64	75.2	70.2	67.6	69.6	70.4	71.1	71.3
	(71.8 to 78.2)	(66.6 to 73.6)	(63.9 to 71.0)	(65.9 to 73.1)	(66.9 to 73.6)	(67.6 to 74.5)	(68.0 to 74.5)
65+	73.9	67.5	70.9	68.5	66.6	64.8	70.3
	(68.7 to 78.6)	(62.4 to 72.3)	(66.1 to 75.2)	(65.4 to 73.0)	(61.3 to 71.4)	(60.2 to 69.2)	(65.5 to 74.8)
Social grade AB	79.7	77.1	79.9	80.9	81.9	81.9	81.7
	(74.9 to 83.8)	(72.2 to 81.4)	(74.4 to 83.0)	(76.1 to 84.9)	(77.3 to 85.8)	(77.8 to 85.5)	(77.6 to 85.2)
Social grade C1C2	71.1	67.8	68.1	67.2	68.8	67.8	69.6
	(67.8 to 74.3)	(64.3 to 71.1)	(64.6 to 71.4)	(63.6 to 70.6)	(65.4 to 72.1)	(63.3 to 70.1)	(66.4 to 72.7)
Social grade DE	57.5	48.9	47.2	43.0	49.2	49.0	48.9
	(52.5 to 62.4)	(44.0 to 53.8)	(42.2 to 52.4)	(38.0 to 48.2)	(44.6 to 54.0)	(44.3 to 53.7)	(47.2 to 51.0)
Per cent of drinkers who are aware of guidelines							
Female	87.8	87.1	89.7	88.4	85.4	87.6	88.1
	(84.5 to 90.5)	(83.8 to 89.8)	(86.6 to 92.2)	(85.1 to 91.1)	(82.0 to 88.3)	(84.4 to 90.2)	(85.2 to 90.6)
Male	82.5	87.0	87.6	88.8	87.5	84.4	88.7
	(79.0 to 85.5)	(83.7 to 89.7)	(84.6 to 90.1)	(85.7 to 91.3)	(84.3 to 90.2)	(81.1 to 87.3)	(85.6 to 91.1)
16–34	81.2	82.5	80.7	84.7	78.9	78.4	83.9
	(76.0 to 85.5)	(77.4 to 86.5)	(75.4 to 85.1)	(79.7 to 88.6)	(73.6 to 83.4)	(73.3 to 82.8)	(79.2 to 87.8)
35–6	86.6	90.6	92.8	90.2	90.7	89.9	90.8
	(83.2 to 89.3)	(87 to 5 to 92.9)	(90.2 to 94.7)	(87 to 1 to 92.6)	(87.9 to 93.0)	(86.8 to 92.4)	(88.0 to 93.0)
65+	86.2	84.5	89.8	89.7	87.3	86.1	88.6
	(81.3 to 90.0)	(79.2 to 88.7)	(85.6 to 92.8)	(85.2 to 92.9)	(82.3 to 91.1)	(81.7 to 89.6)	(84.2 to 91.8)
Social grade AB	93.1	92.9	95.0	95.5	93.5	94.3	92.3
	(89.1 to 95.7)	(89.1 to 95.4)	(91.9 to 96.9)	(91.9 to 97.6)	(89.9 to 95.9)	(91.2 to 96.3)	(88.7 to 94.8)
Social grade C1C2	86.0	89.2	89.1	87.3	85.3	84.4	90.2
	(82.8 to 88.7)	(86.0 to 91.7)	(86.1 to 91.6)	(84.1 to 90.0)	(81.8 to 88.2)	(80.9 to 87.3)	(87.4 to 92.4)
Social grade DE	69.6	70.2	74.9	77.8	76.8	74.4	75.4
	(62.9 to 75.6)	(63.3 to 76.3)	(67.9 to 80.8)	(70.8 to 83.6)	(70.5 to 82.1)	(67.7 to 80.1)	(68.8 to 81.0)
Low risk (<14 units per week)	88.3	87.9	88.6	92.4	88.1	88.5	90.4
	(85.5 to 90.7)	(85.1 to 90.2)	(85.9 to 90.8)	(89.9 to 94.3)	(85.3 to 90.4)	(85.8 to 90.7)	(87.8 to 92.5)
Increasing/high risk (14+units per week)	91.7	96.5	96.0	97.2	94.3	88.0	95.0
	(84.4 to 95.6)	(90.3 to 97.3)	(93.7 to 98.2)	(87.7 to 95.9)	(84.1 to 93.2)	(91.1 to 97.9)	(92.3 to 95.0)
Per cent of drinkers saying guideline was 14 units per week or less							
Female	51.9	55.8	61.6	59.1	55.4	54.4	57.4
	(47.0 to 56.7)	(51.1 to 60.4)	(57.1 to 66.0)	(54.3 to 63.7)	(50.9 to 60.0)	(49.8 to 59.0)	(53.0 to 61.6)
Male	24.3	22.6	**43.3**	**35.0**	**32.7**	**36.1**	**35.6**
	(20.5 to 28.4)	(18.9 to 26.7)	**(38.9** to **47.8)**	**(30.5** to **39.9)**	**(28.7** to **36.8)**	**(32.0** to **40.4)**	**(31.6** to **39.9)**
16–34	37.7	37.5	45.0	43.1	37.4	33.1	42.9
	(31.8 to 43.9)	(31.5 to 44.0)	(39.0 to 51.2)	(36.7 to 49.7)	(31.8 to 43.2)	(27.8 to 38.8)	(37.1 to 48.9)
35–64	36.4	39.8	**55.8**	**49.8**	46.3	**49.7**	48.6
	(31.8 to 41.2)	(35.3 to 44.5)	**(51.0** to **60.5)**	**(44.8** to **54.8)**	(41.8 to 50.9)	**(45.0** to **54.3)**	(44.2 to 52.9)
65+	40.7	36.8	**52.8**	44.1	45.0	48.3	44.8
	(34.2 to 47.4)	(30.6 to 43.4)	**(46.9** to **58.7)**	(38.0 to 50.3)	(38.7 to 51.4)	(42.5 to 54.1)	(38.9 to 50.9)
Social grade AB	43.4	46.0	55.7	53.7	49.6	54.0	48.1
	(36.9 to 50.2)	(39.9 to 52.2)	(49.7 to 61.5)	(47.3 to 60.0)	(43.7 to 55.6)	(48.3 to 59.6)	(42.7 to 53.7)
Social grade C1C2	37.3	37.2	**53.0**	45.6	41.6	42.9	**50.3**
	(33.1 to 41.7)	(32.8 to 41.7)	**(48.6** to **57.4)**	(41.1 to 50.2)	(37.4 to 45.9)	(38.5 to 47.4)	**(46.0** to **54.5)**
Social grade DE	29.4	29.1	42.4	35.4	36.8	31.9	30.2
	(23.2 to 36.4)	(22.8 to 36.2)	(35.0 to 50.2)	(28.1 to 43.5)	(30.6 to 43.5)	(25.8 to 38.7)	(23.9 to 37.3)
Low risk (<14 units per week)	41.8	42.2	**55.1**	**50.8**	48.8	48.6	50.0
	(37.6 to 46.0)	(38.1 to 46.4)	**(51.2** to **58.9)**	**(46.6** to **55.0)**	(44.7 to 52.8)	(44.6 to 52.6)	(46.1 to 53.9)
Increasing/high risk (14+units per week)	29.2	28.7	**50.7**	45.8	35.2	40.3	43.2
	(22.5 to 38.2)	(22.3 to 36.9)	**(40.8** to **56.4)**	(35.5 to 51.6)	(26.1 to 40.0)	(33.0 to 46.7)	(35.0 to 49.5)

*All figures are percentages with 95% CIs in parentheses unless otherwise stated. Bold text indicates significant differences compared with December 2015 based on 95% CIs.

**Figure 1:**
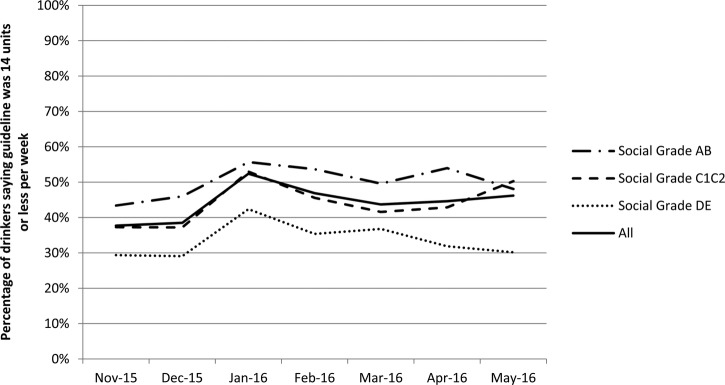
Knowledge of UK lower risk drinking guidelines by social grade.

Those in grade DE were also significantly less likely to say the guideline was 14 units or less than those in grade AB (29% vs 46%). This gap was still present in January despite both groups registering the change in guidelines (42% vs 56%) and persisted in May after knowledge decreased again (30% vs 48%; figure 1). Only the guideline for men was changed in January and the change in knowledge is specific to men. While 23% of men and 56% of women said the guideline was 14 units or less in December, the proportion of men saying this increased significantly to 43% in January but only changed slightly to 62% for women.

**Figure 2:**
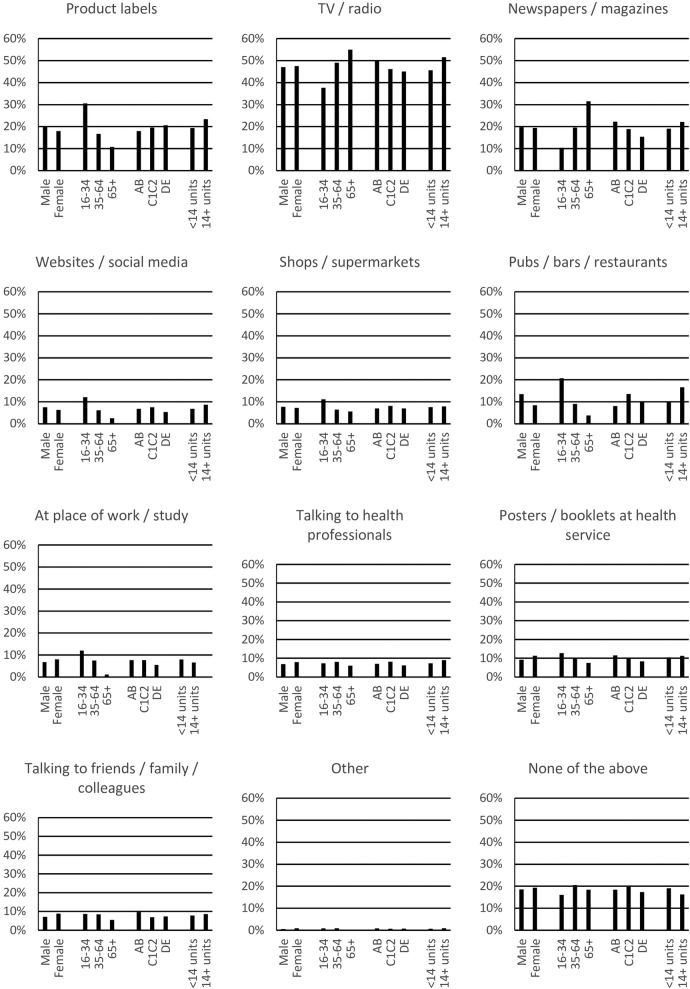
Exposure in last month by subgroup among drinkers who gave a figure for the guidelines.

Among those who gave a figure for the guidelines, 16–34 years old were more likely than older drinkers to report exposure via product labels, websites and social media, or at pubs, bars, restaurants or their place of work or study. In contrast, older drinkers were more likely to report exposure via TV, radio, newspapers or magazines (figure 2). Differences between other population groups were not sufficiently large to be of major policy significance (ie, <10 percentage points).

## Discussion

The publication of revised UK lower risk drinking guidelines in January 2016 did not increase awareness of the existence of drinking guidelines. This may partly reflect high baseline awareness; however, there were also no increases in subpopulations where baseline awareness was lower (eg, drinkers in social grade DE). Although the new guidelines of 14 units a week were a change from daily to weekly guidance, only the male guideline was actually reduced and the proportion of male drinkers saying the guideline was 14 units per week or less increased following the announcement, although only to 43%. This figure declined again after January, although there was some evidence of an emerging secondary gradual increase in knowledge. As with awareness, drinkers in social grade DE had lower levels of knowledge before, during and after January than those in higher social grades. The lack of large-scale promotional activity beyond news coverage meant that television and radio were the main media through which drinkers were exposed to the drinking guidelines and this was particularly the case after the announcement in January. Less than a quarter of drinkers reported hearing about the guidelines through any other medium in January.

This study is the first to the authors' knowledge to use prospective high-frequency survey data to examine the emergence and decay of short-term effects of promoting new or revised drinking guidelines. Further strengths include the use of consistent data collection methods and measures over survey waves, the inclusion of preintervention and postintervention data, the nationally representative sample and the examination of multiple outcomes including awareness, knowledge and place of exposure. Limitations include the short preintervention period and the potential for the traditionally heavy and light drinking months of December and January to confound intervention effects. With regard to exposure, the accessibility of television clips and newspaper reports through social media and websites means that the source of some exposure may be difficult for respondents to classify. Finally, self-reporting biases are common to all studies on alcohol use and lead to underestimation of alcohol consumption.^26^ This will affect accurate classification of respondents into consumption groups for subgroup analyses but is unlikely to impact the main outcome measures. Short-term effects of promoting drinking guidelines on alcohol consumption and related harm are not examined in this paper as these outcomes are the focus of an ongoing longer term evaluation. The findings arise from a nationally representative sample of drinkers living in private households in England. Therefore, it is reasonable to assume these findings are generalisable to other high-income countries with comparable drinking cultures after allowing for baseline differences in outcome measures; however, data on these baseline differences are scarce.

Overall, the findings broadly align with previous studies by suggesting that announcing revisions to drinking guidelines can lead to modest improvements in drinkers' knowledge of the guidelines.^16–18^ However, our results additionally suggest that without more extensive or sustained promotional efforts, knowledge remains low, any gains in knowledge may be short-lived, and social inequalities persist in the awareness and knowledge, which the UK Government regards as necessary “for people to make responsible and informed choices about their drinking” ref. 2, p. 27.

To date, the UK Government has not announced a major promotional campaign for the new drinking guidelines. This is likely to limit their impact as routine promotional activity appears to go largely unnoticed. Only a small minority of drinkers reported recent exposure to the guidelines from sources not linked to news coverage. In particular, only around a fifth of drinkers noticed the guidelines on product labels despite alcohol producers and retailers ensuring around 80% of products include the drinking guidelines on their label as part of the Public Health Responsibility Deal (PHRD).^27^ The reason this information has failed to register with drinkers is unclear as previous literature has suggested such labelling can be effective in enabling drinkers to track their alcohol intake and conform to drinking guidelines.^28^ However, an evaluation of the PHRD noted that the UK guidelines were typically presented on the bottom of the reverse label of products and in font sizes smaller than those recommended for easy readability.^29^

Further research is required to evaluate how revision and promotion of the UK's lower risk drinking guidelines affects alcohol consumption and alcohol-related harm. The results above suggest a rapidly decaying short-term effect on knowledge but also indicate a secondary effect may be emerging and research will be required to characterise the trajectory of any effects in the medium and long term. Qualitative evidence is also required regarding how drinkers accommodate the new guidelines within their existing understanding about alcohol-related risks and apply that broader understanding to their own and others' alcohol consumption. Lovatt *et al*^30^ have described how drinkers used lay epidemiology to interpret the previous guidelines and further work in this vein may be profitable and should take account of how the guidelines were presented to the public by health professionals, news outlets and other public figures, both supportive and critical.

## Conclusions

Publication of new or revised lower risk drinking guidelines can improve drinkers' knowledge of these guidelines within all sociodemographic groups; however, in the absence of sustained promotional activity, positive effects may be short-lived and social inequalities in awareness and knowledge of guidelines are likely to persist.
