# Supplementary material for: Short-term effects of announcing revised lower risk national drinking guidelines on related awareness and knowledge: a trend analysis of monthly survey data in England
Source: BMJ Open. 2016 Dec 1;6(12):e013804. doi: 10.1136/bmjopen-2016-013804 (PMC5168688; doi:10.1136/bmjopen-2016-013804)
Supplement: Supplementary appendix [file bmjopen-2016-013804supp_appendix.pdf]

Table A1: Trends in main outcome measures by survey month

	Nov. 2015	Dec. 2015	Jan. 2016	Feb. 2016	Mar. 2016
**Drinker status: Base - All respondents (N)**	1,689	1,660	1,712	1,674	1,679
Drinkers (%)	71.2*	65.9	66.1	65.2	67.7*
**Awareness: Base - All drinkers (N)**	1,102	1,035	1,103	1,037	1,109
Heard of guidelines (%)	85.1*	87.1	88.6	88.6	86.5
**Knowledge of new guideline: Base - All drinkers (N)**	1,104	1,035	1,111	1,040	1,119
Below 14 units per week/two units per day (%)	18.3	17.9	23.4	19.8	19.3
14 units per week/two units per day (%)	19.4	20.6	29.0	27.1	24.4
Above 14 units per week/two units per day (%)	32.0	33.2	22.1***	29.7***	27.7***
Not aware of drinking guidelines (%)	14.9	13.0	11.4***	11.4**	13.5*
Aware of drinking guidelines but doesn't know it (%)	15.4	15.3	14.2**	12.1***	15.1
**Exposure in last month: Base - Drinkers who gave a figure for the guideline (N)** *Multiple responses permitted*	771	742	822	786	804
Product labels (%)	20.8	19.4	17.8	14.3**	21.0
TV/radio (%)	35.8	32.5	64.8***	54.0***	50.9***
Newspapers/magazines (%)	16.5	15.0	24.3***	22.9***	20.5***
Websites/social media (%)	5.7	6.3	7.5	5.3	8.8
Shops/supermarkets (%)	8.6	8.0	6.7	7.7	7.9
Pubs/bars/restaurants (%)	13.2	12.8	11.3	10.1*	11.2
At place of work/study (%)	7.1	6.0	7.2	8.0	8.1
Talking to health professionals (%)	10.8**	6.9	6.9	5.3	8.1
Posters/booklets at health service (%)	11.5	10.6	9.6	9.0	12.0
Talking to friends/family/colleagues (%)	7.6	5.1	9.2**	8.3	8.9
Other (%)	1.5	1.2	0.3	0.6	0.4
None of the above (%)	26.4**	32.3	7.4***	13.3***	15.5***

* p<0.10, ** p<0.05, *** p<0.01. Reference category is December 2015 in all binary logistic regressions and December 2015 and 'Above 14 units per week/two units per day' for the multinomial logistic regression on knowledge.

Table A2: Trends in main outcome measures within sociodemographic groups by survey month

	Nov. 2015	Dec. 2015	Jan. 2016	Feb. 2016	Mar. 2016
**Unweighted number of cases (N)**					
Female (ref)	805	820	848	817	823
Male	884	840	871	860	866
16-34 (ref)	546	522	505	518	502
35-64	759	738	759	725	784
65+	384	400	455	434	403
Social grade AB (ref)	332	376	416	366	371
Social grade C1C2	893	832	876	856	845
Social grade DE	464	452	427	455	473
Non-drinker	585	625	608	637	570
Low risk[1]	677	644	744	648	675
Increasing risk	149	162	172	173	171
High risk	29	27	17	22	29
**% of sample who are drinkers**					
Female (ref)	66.9	61.9	62.3	62.7	63.2
Male†††	73.8	69.9	70.4	68.2	73.0
16-34 (ref)	60.1	57.9	60.8	56.2	64.5
35-64†††	75.2	70.2	67.6*	69.6	70.4
65+†††	73.9	67.5	70.9	68.5	66.6
Social grade AB (ref)	79.7	77.1	79.9	80.9	81.9
Social grade C1C2†††	71.1	67.8	68.1	67.2	68.8
Social grade DE†††	57.5*	48.9	47.2	43.0*	49.2
**% of drinkers who are aware of guidelines:**					
Female (ref)	87.8	87.1	89.7	88.4	85.4
Male	82.5	87.0	87.6	88.8	87.5
16-34 (ref)	81.2	82.5	80.7	84.7	78.9
35-64†	86.6	90.6	92.8	90.2*	90.7
65+	86.2	84.5	89.8	89.7	87.3
Social grade AB (ref)	93.1	92.9	95.0	95.5	93.5
Social grade C1C2	86.0	89.2	89.1	87.3	85.3
Social grade DE†††	69.6	70.2	74.9	77.8	76.8
Low risk (ref)	88.3	87.9	88.6	92.4	88.1
Increasing risk††	91.7	96.5	96.0	97.2	94.3
High risk	97.3	84.2	97.8	92.1	83.4
**% of drinkers saying guideline was 14 units per week/two units per day or less**					
Female (ref)	51.9	55.8	61.6	59.1	55.4
Male†††	24.3	22.6	43.3***	35.0	32.7**
16-34 (ref)	37.7	37.5	45.0	43.1	37.4
35-64	36.4	39.8	55.8	49.8	46.3
65+	40.7	36.8	52.8	44.1	45.0
Social grade AB (ref)	43.4	46.0	55.7	53.7	49.6
Social grade C1C2	37.3	37.2	53.0	45.6	41.6
Social grade DE†††	29.4	29.1	42.4	35.4	36.8
Low risk (ref)	41.8	42.2	55.1	50.8	48.8
Increasing risk	29.2	28.7	50.7*	45.8	35.2
High risk	33.6	31.9	28.0	23.6	16.6*

[1]Low risk (less than 14 units per week), Increasing risk (14 to less than 35 units per week for females, 14 to less than 50 units per week for males), High risk (35+ units per week for females and 50+ units per week for males).
† p<0.10, †† p<0.05, †††p<0.01. Significance of the main effect for this characteristic in multivariate regression models with the top category the reference.
* p<0.10, ** p<0.05, *** p<0.01. Significance in multivariate regression models of the interaction between characteristic and month with the top category of the characteristic and December 2015 the reference in each case.
